# Health-disease processes among women agriculturalists in Central Amazon: work and environmental vulnerabilities

**DOI:** 10.1590/0102-311XEN098324

**Published:** 2026-01-09

**Authors:** Letícia Souza Reis, Taciana Lemos Barbosa, Eduardo Capanema, Lucas Ferrante, Socorro de Fátima Moraes Nina

**Affiliations:** 1 Programa de Pós-graduação em Psicologia, Universidade Federal do Amazonas, Manaus, Brasil.; 2 Instituto de Saúde Coletiva, Universidade Federal da Bahia, Salvador, Brasil.; 3 Universidade do Estado do Amazonas, Manaus, Brasil.; 4 Universidade Federal de Minas Gerais, Belo Horizonte, Brasil.; 5 Laboratório da Evolução e Genética Animal, Universidade Federal do Amazonas, Manaus, Brasil.; 6 Escola de Artes, Ciências e Humanidades, Universidade de São Paulo, São Paulo, Brasil.

**Keywords:** Environmental Health, Environment and Public Health, Environmental Psychology, Environmental Science, Gender, Saúde Ambiental, Meio Ambiente e Saúde Pública, Psicologia Ambiental, Ciência Ambiental, Gênero, Salud Ambiental, Medio Ambiente y Salud Pública, Psicología Ambiental, Ciencia Ambiental, Género

## Abstract

This study examined the health-disease process among women agriculturalists in traditional communities of the Central Amazon, focusing on the intersections between labor, environmental conditions, and social factors. Fieldwork was conducted in the Rio Negro Sustainable Development Reserve, using semistructured interviews and participant observation across five communities. Content analysis was validated by rarefaction and word co-occurrence techniques, confirming the adequacy of the sample and the obtained thematic categories. Results indicate that agricultural work contributes to women’s health, autonomy, and dignity, while also exposing them to physical risks such as accidents and bodily strain. Care practices centered around traditional remedies and limited access to primary healthcare, which is hampered by long distances and resource shortages. Land conflicts, illegal land grabbing, and the impacts of major infrastructure projects, such as the Rio Negro Bridge and the proposed roads, further undermine healthcare provision. This study concludes that the multifactorial health-disease process is deeply tied to living and working conditions within a broader socioenvironmental context. Strengthening primary care and safeguarding traditional territories are essential to ensuring comprehensive health for these populations.

## Abstract (pt)

Este estudo examinou o processo saúde-doença entre mulheres agricultoras em comunidades tradicionais da Amazônia Central, com foco nas interseções entre trabalho, condições ambientais e fatores sociais. O trabalho de campo foi conduzido na Reserva de Desenvolvimento Sustentável do Rio Negro, utilizando entrevistas semiestruturadas e observação participante em cinco comunidades. A análise de conteúdo foi validada por meio de técnicas de rarefação e coocorrência de palavras, confirmando tanto a adequação da amostra quanto as categorias temáticas. Os resultados indicam que o trabalho agrícola contribui para a saúde, autonomia e dignidade das mulheres, ao mesmo tempo em que as expõe a riscos físicos, como acidentes e esforços excessivos. As práticas de cuidado são centradas no uso de remédios tradicionais, juntos com acesso limitado à atenção primária à saúde, que é dificultado por longas distâncias e a escassez de recursos. A provisão de serviços de saúde é ainda mais comprometida por conflitos de terra, apropriação ilegal de terras e os impactos de grandes projetos de infraestrutura, como a Ponte do Rio Negro e as estradas propostas. O estudo conclui que o processo saúde-doença é multifatorial e profundamente ligado às condições de vida e trabalho dentro de um contexto socioambiental mais amplo. O fortalecimento da atenção primária e a proteção dos territórios tradicionais são essenciais para garantir a saúde integral dessas populações.

## Abstract (es)

Este estudio examinó el proceso salud-enfermedad entre las mujeres agricultoras de las comunidades tradicionales de la Amazonia Central, con énfasis en las intersecciones entre el trabajo, las condiciones ambientales y los factores sociales. El trabajo de campo se llevó a cabo en la Reserva de Desarrollo Sostenible del Río Negro, mediante entrevistas semiestructuradas y observación participante en cinco comunidades. El análisis de contenido se validó mediante técnicas de rarefacción y coocurrencia de palabras, lo que confirmó tanto la pertinencia de la muestra como las categorías temáticas. Los resultados indican que el trabajo agrícola contribuye a la salud, la autonomía y la dignidad de las mujeres, exponiéndolas al mismo tiempo a riesgos como accidentes y esfuerzo físico. Las prácticas de atención sanitaria se centran en el uso de remedios tradicionales, combinados con un acceso limitado a la atención primaria, que se ve obstaculizado por las largas distancias y la escasez de recursos. La prestación de servicios de salud se ve aún más afectada por los conflictos territoriales, la apropiación ilegal de tierras y los impactos de grandes proyectos de infraestructura, como el puente sobre el Río Negro y las carreteras en proyecto. El estudio concluye que el proceso salud-enfermedad es multifactorial y está profundamente unido a las condiciones de vida y de trabajo dentro de un contexto socioambiental más amplio. El fortalecimiento de la atención primaria y la protección de los territorios tradicionales son esenciales para garantizar la salud integral de estas poblaciones.

## Introduction

Traditional communities in the Central Amazon are more vulnerable to diseases due to genetic factors and the absence of medical professionals and supplies ^1^ ^,^ ^2^ ^,^ ^3^ ^,^ ^4^. The wealth circulating in the region fails to contribute to the reduction of hardships and social inequalities, which are even more profound in Indigenous and rural communities in the Amazon ^4^. These traditional and rural populations greatly depend on the public health system ^4^. Ethnic disparities in public health among traditional populations in Brazil have worsened in the past four years due to a series of measures adopted by the government of President Jair Bolsonaro (2019-2022) ^3^ ^,^ ^4^ ^,^ ^5^, invasions of the territories of these populations ^6^ ^,^ ^7^ ^,^ ^8^ ^,^ ^9^, and the COVID-19 pandemic ^3^ ^,^ ^10^ ^,^ ^11^ ^,^ ^12^ ^,^ ^13^, during which the Federal Government denied potable water, medical supplies, and basic assistance to traditional peoples in Brazil, especially in the Amazon, such as the Indigenous, riverine people (*ribeirinhos*), and *quilombolas* ^3^ ^,^ ^5^ ^,^ ^10^ ^,^ ^14^. Basic healthcare was also compromised with the abandonment of over 8,000 physicians who served these communities in the interior of the country, mainly in the Amazon ^3^. Despite a change in government in 2023 with the promise of a more technical and environmentally engaged administration, the reality has shown an increase in threats to traditional territories (such as large infrastructure projects) and a lackluster improvement in health, including inadequate vaccination strategies for the vulnerable population of the Amazon ^15^.

The violation of the rights of these peoples and their territories has advanced as part of a “progressive” agenda ^6^ ^,^ ^16^ ^,^ ^17^ ^,^ ^18^ and often fueled by politicians’ hate speech, intensifying invasions into these territories and weakening the environment, way of life, and work of these communities, especially subsistence agriculture ^19^ ^,^ ^20^. This set of factors that exacerbated health disparities in traditional communities has primarily affected women ^21^ ^,^ ^22^ since their role in society is often unacknowledged or treated as secondary ^23^. The environmental and territorial conditions in which they live - particularly those related to access to land, forests, and water resources ^21^ ^,^ ^24^ - deeply shape rural women’s everyday lives. These territorial characteristics influence economic production and social reproduction processes (understood here as the set of activities and relationships that sustain life, such as caregiving, food production, and community organization) ^21^ ^,^ ^25^ ^,^ ^26^. In this context, rural women develop distinctive forms of health and life care practices that emerge from their lived experiences and are closely tied to demands for public and universal health services ^4^ ^,^ ^21^ ^,^ ^27^. Understanding the intersections between labor conditions, the health-disease process, and environmental factors in the lives of rural women is a complex task, particularly due to the lack of disaggregated official data by place of residence or specific work environments ^21^. This lack of granularity makes it difficult to understand how different environments - such as domestic settings, agricultural fields, or forest areas - affect women’s health. The term “health-disease process” refers to a dynamic and contextual understanding of health encompassing access to healthcare and the social, economic, and environmental factors that influence how illness is produced, perceived, and managed in daily life. In the case of rural women, this process is deeply shaped by their living and working conditions, which expose them to a range of physical and psychosocial risks and vulnerabilities that contribute to illness, suffering, and broader health grievances ^27^ ^,^ ^28^ ^,^ ^29^ ^,^ ^30^. Given the complexity of factors affecting lifestyles in the Amazon and rural women, this study analyzed the health-disease process in women agriculturalists in traditional communities in the Central Amazon and its relationship with work and the vulnerability of the environment.

## Methods

### Study area

**Figure 1:**
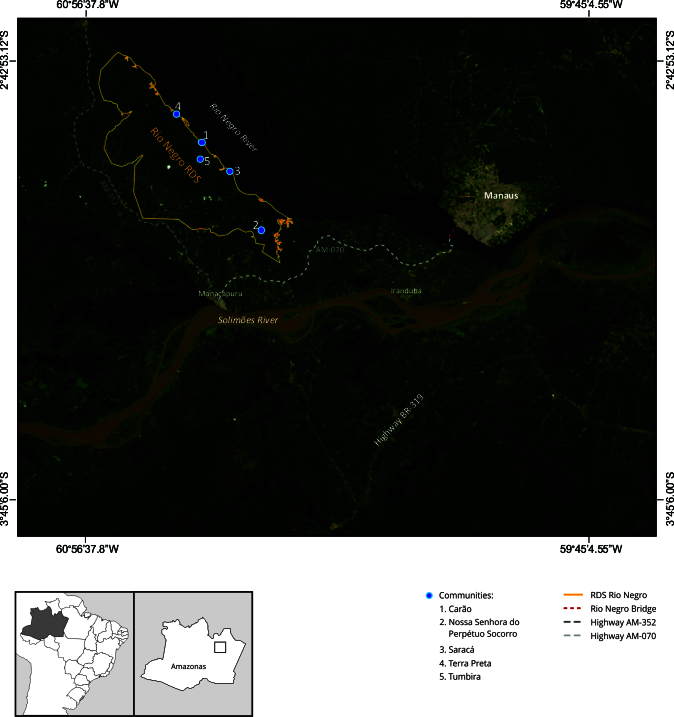
Map of the Rio Negro Sustainable Development Reserve (RDS) in the state of Amazonas, Brazil, showing the location of its five study communities.

Note: the map highlights the boundaries of the RDS Rio Negro (yellow line), the Rio Negro Bridge (red line), and the main access highways (AM-070 and AM-352) connecting the reserve to Manaus and neighboring municipalities.

The Rio Negro Sustainable Development Reserve (RDS, acronym in Portuguese) (Figure 1) constitutes a protected area of 102,978.83 hectares located in the state of Amazonas, Brazil ^31^. It spans three municipalities: Iranduba, Novo Airão, and Manacapuru (comprising 80%, 16%, and 4% of the reserve, respectively) ^31^. The RDS Rio Negro encompasses 19 traditional riverine communities, including Nossa Senhora do Perpétuo Socorro, Tumbira, Saracá, Terra Preta, among others ^31^. Its creation in 2008 aimed to safeguard biodiversity ^32^ and support the livelihoods of local populations by the sustainable use of natural resources ^33^. Its main economic activities include small-scale agriculture - focused on manioc, bananas, and vegetables - and extractive practices such as fishing and the collection of non-timber forest products. This context provides a vital backdrop for examining the experiences and challenges faced by women agriculturalists within the reserve.

The region experiences climatic dynamics that include a seasonal rainfall pattern, with a rainy season and a dry season, directly influencing the lives of local communities and their movements ^34^. The reserve faces significant threats to its residents and their way of life, including the pressure of deforestation, illegal mining, predatory hunting and fishing, and land invasions ^35^. Climate change also poses a growing challenge, with extreme weather events such as droughts isolating communities during the Amazonian summer ^36^ and impacting biodiversity ^37^.

### Target ethnic group

The women rural workers from the riverine communities of the RDS Rio Negro in the Central Amazon show an impressive ethnic diversity, reflecting its cultural richness. Many have Indigenous origins, belonging to ethnic groups such as Tukano, Baré, Baniwa, Tuyuca, and Mura, in contrast with the intermingling of Indigenous people with European colonizers. Their way of life is deeply rooted in nature and adaptation to natural resources. They master traditional farming, fishing, and craft techniques, utilizing their ancestral knowledge to ensure the subsistence of their families ^21^ ^,^ ^25^. Additionally, these women serve as guardians of their communities, playing a central role in maintaining social cohesion and transmitting traditional knowledge. They are responsible for the care and well-being of their families and for preserving cultural practices, stories, and rituals. Via this stewardship, they pass on a deep respect for the forest, rivers, and other natural elements to younger generations ^4^ ^,^ ^11^. Their daily labor and profound relationship with the Amazon environment reflect the ethnic diversity and cultural resilience that shape the identity of riverine communities in the region.

### Field data collection

To assess the health-disease process among women agriculturalists in the RDS Rio Negro (Figure 1), semistructured interviews (Box 1) and participant observation were conducted with five women agriculturalists from five communities across the reserve: (1) Carão, (2) Nossa Senhora do Perpétuo Socorro, (3) Saracá, (4) Terra Preta, and (5) Tumbira (Figure 1). This geographical scope enhanced the representativeness of the sample, ensuring that perspectives from across the entire RDS Rio Negro territory were captured and minimizing biases that might arise from focusing on a single community.

Semistructured interviews consist of guided conversations based on key topics, offering the flexibility to explore issues in greater depth as they arise (Box 1). On the other hand, participant observation enables immersive engagement so researchers can observe and interact with participants in their everyday environments. All semistructured interviews were conducted in April 2022. By combining these complementary methods, we ensured a more nuanced understanding of the lived experiences of women in the RDS Rio Negro. Additionally, we conducted a thorough review of bibliographical and documentary sources, incorporating testimonies from residents of four communities within the reserve to further triangulate and contextualize our findings.

**Box 1:** Semistructured interview guide.

SECTION	QUESTION
a. Identification	Name/Codename: ____; Age: ____; Marital status: ____; Occupation: ____; Time in current job: ____.
	Where were you born? Do you have children? How many? What is your income (daily/monthly)? How long have you lived in this community?
	At what age did you get married (if applicable)? At what age did you have your first child (if applicable)?
	Did you complete your studies? If not, up to which grade did you study? Why did you stop studying?
b. Health-disease process	In your opinion, what does being healthy mean? What do you do when you are ill? Do you experience any discomfort while working? If so, what kind?
c. Meaning of work	What does work mean to you? Do you think there is a division between men’s and women’s work?
	Do you feel recognized for your work in family farming? Do you believe your work is important?
	Do you think the work of female farmers is valued? What do you consider to be the main risks in your work?
	Have you ever had a work-related accident? What challenges do you face in carrying out your work?
	How do you cope with these challenges?
d. Relationship with the environment	How do you feel about the place where you live?

Note: this box shows the adapted semistructured interview questions for women agriculturalists in the Rio Negro Sustainable Development Reserve. The questions are designed to explore their health-disease processes, perceptions of work, and relationship with the environment.

### Analyses

**Figure 2:**
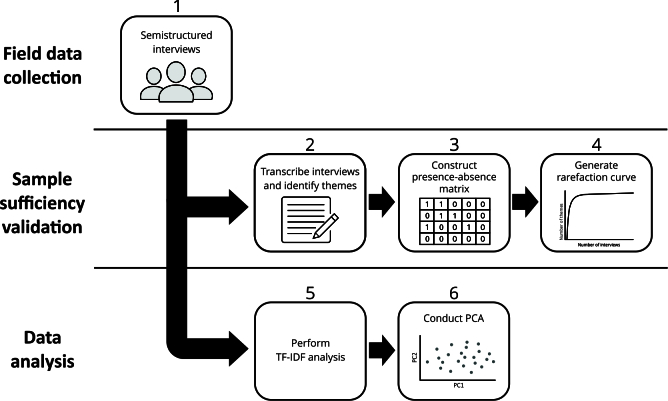
Methodological workflow, including field data collection, sample sufficiency validation, and data analysis.

PCA: principal component analysis; TF-IDF: term frequency-inverse document frequency. Note: steps include conducting semistructured interviews (1), transcribing and finding themes (2), building a presence-absence matrix (3), generating a rarefaction curve (4), performing TF-IDF analysis (5), and conducting PCA (6) to validate and explore thematic structures.

To assess whether the number of conducted semistructured interviews sufficiently captured the thematic diversity of the investigated phenomenon, a rarefaction analysis was performed ^38^ ^,^ ^39^. Each interview was transcribed and subjected to a thematic content analysis ^40^, which found the themes in each interview. Based on this analysis, a binary presence-absence matrix was constructed in which each row represented an individual interview and each column corresponded to a distinct theme; values of 1 or 0 indicated the presence or absence of each theme in an interview, respectively. This matrix served as the input dataset for the rarefaction analysis (Figure 2).

Rarefaction curves were generated on EstimateS, version 9.1.0 (https://www.robertkcolwell.org/pages/1407-estimates) ^41^, with 1,000 randomizations using the bootstrap estimator. These curves plot the cumulative number of unique themes as a function of the number of interviews analyzed (Figure 2). As the curve approaches an asymptote, the addition of further interviews yields diminishing returns regarding novel thematic content. Thus, the stabilization of the curve indicates that the sample size captured the thematic variability of the studied phenomenon ^38^ ^,^ ^39^.

To validate the thematic structure from the content analysis, a computational word co-occurrence analysis was implemented based on the raw textual data. This process involved combining term frequency-inverse document frequency (TF-IDF) ^42^ ^,^ ^43^ and principal component analysis (PCA) ^44^ ^,^ ^45^ ^,^ ^46^, which enabled us to empirically assess the robustness of the categories and their key topics (Figure 2).

The TF-IDF analysis was conducted using the scikit-learn library. First, all interview transcripts were tokenized, and punctuation was removed. Stopwords - terms with minimal semantic value - were excluded ^42^ ^,^ ^43^. Part-of-speech tagging was then applied to find the relevant nouns, including singular and plural forms. A new document was created with these relevant lexical units, which served as input for the TF-IDF model (Figure 2). The TF-IDF algorithm assigned a score to each noun based on its relative frequency within a given document (TF) and its rarity across the full set of interviews (IDF), producing a ranked list of terms in descending order of relevance ^42^ ^,^ ^43^.

Subsequently, the nouns ranked by TF-IDF scores ^42^ ^,^ ^43^ were used as input variables for a PCA ^44^ ^,^ ^45^ ^,^ ^46^, a dimensionality reduction technique that enables the visualization of high-dimensional data by transforming correlated variables into a set of uncorrelated principal components ^45^ ^,^ ^46^. This method (Figure 2) was applied to explore the clustering and distribution of themes that had been defined during content analysis, providing an additional layer of validation for the analytical categories ^44^ ^,^ ^45^ ^,^ ^46^.

## Results

### Sample sufficiency and content analysis validation

**Figure 3:**
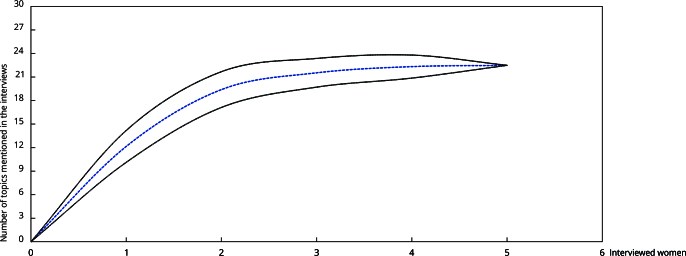
Rarefaction curve illustrating the cumulative number of unique topics in the interviews as a function of the number of interviewed women.

Note: the curve reaches an asymptote, indicating sample sufficiency and thematic saturation. Shaded lines represent confidence intervals based on 1,000 bootstrap randomizations.

A rarefaction curve indicated adequate topic sampling via interviews as the curve reached an asymptote, suggesting that the interviews captured most of response diversity (Figure 3). Additionally, the stability of the curve indicates consistency in the results from interviews during topic sampling (Figure 3).

**Figure 4:**
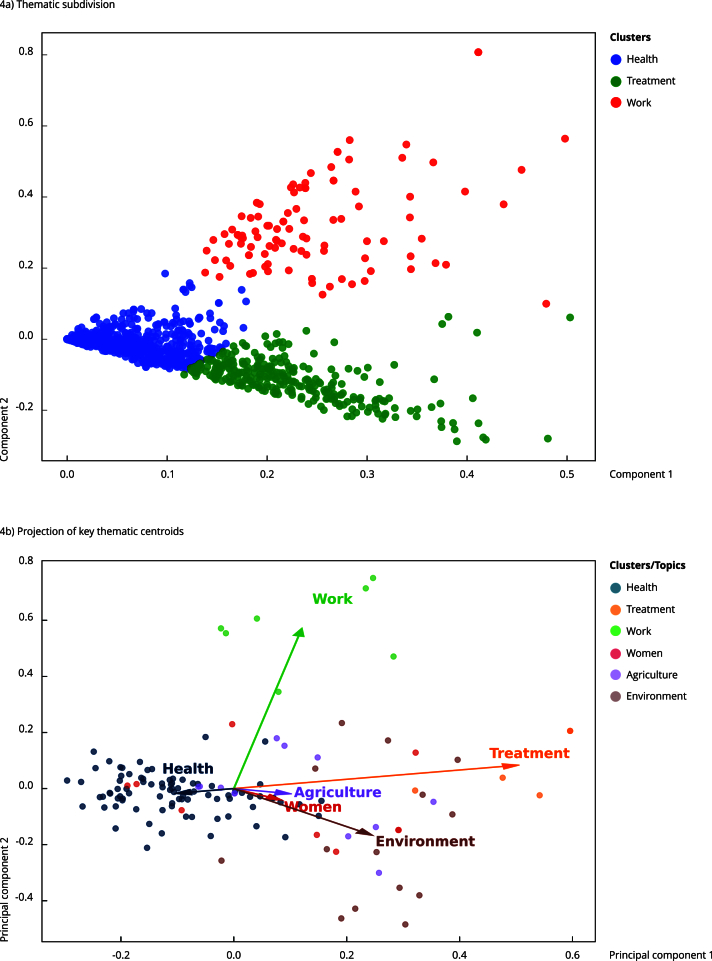
Principal component analysis of the term frequency-inverse document frequency (TF-IDF) results.

Note: 4a - thematic subdivision of textual data showing well-defined statistical clusters corresponding to the categories health (blue), treatment (green), and work (red), indicating consistent internal cohesion and distinction among themes. 4b - projection of key thematic centroids (health, treatment, work, woman, agriculture, and environment) showing semantic distances and interrelationships between broader categories.

The thematic clustering analysis in Figure 4a (resulting from the PCA applied to the TF-IDF data) showed three statistically distinct clusters: “Health” (blue), “Treatment” (green), and “Work” (red). These clusters highlight a clear and cohesive thematic structure in the women’s statements, indicating that although the concepts are semantically related, their articulation show noticeable distinctions (Figure 4a).

These results can be interpreted considering the interviewees’ accounts, according to which the idea of “being healthy” is intrinsically tied to the ability to work and access to specific treatments. However, the statistical separation between the “Treatment” and “Work” clusters shows that, while both are components of the health experience, they are referenced in different ways (Figure 4a). This separation suggests that participants deem treatment needs as distinct demands, often linked to the absence of adequate public policies and the difficulty of accessing specialized medical care - elements that differ from the more everyday structural role work (especially in subsistence) plays in maintaining health. The proximity of the themes within the principal components also reinforces the complexity of health perception in women in riverine communities, for whom health - rather than an isolated concept - is interdependent on other structural factors such as work, environment, and access to healthcare services (Figure 4a).

In Figure 4b, the projection of thematic centroids in the principal component space enables us to visualize the semantic distances between the main topics the interviewees discussed: “Health”, “Treatment”, “Work”, “Women”, “Agriculture”, and “Environment”. The vector position of these themes suggests that “Health” occupies a central articulating role, being closer to the axes representing “Women” and “Agriculture”. This indicates that their discourse often constructs health based on experiences related to womanhood and subsistence agricultural activities (Figure 4b).

The proximity between the vectors “Women” and “Agriculture” also highlights the central role of women as food producers and caretakers of their families, reinforcing the direct link between agricultural work and the maintenance of family health (Figure 4b). The “Environment” vector, although related to and encompassing vectors such as “Women” and “Agriculture”, is more distant from the other themes, suggesting that environmental issues form a broader discourse field that extends beyond the daily notions of health, gender, and agriculture (Figure 4b). This includes connections to land, water, and the territories themselves, which are threatened by land conflicts and speculation.

On the other hand, the “Treatment” vector shows a strong loading on the first principal component, indicating a more specific and autonomous semantic field related to healthcare infrastructure and access to medical services - not necessarily tied to everyday work or environmental practices. Meanwhile, the “Work” vector stands out along the second principal component, reaffirming its connection to physical effort, productive activity, and women’s social role (Figure 4b). Our results indicate that participants perceive agriculture more as a way of life, shaped by gender and environmental contexts, rather than simply as a form of work, as evinced by the PCA (Figure 4b). These visual representations (Figure 4b) confirm the centrality of health as a relational category - constructed by its connections to gender, production, and environmental context - and show the specificity of certain themes, such as medical treatment, which emerge as distinct needs, although fundamental to the full realization of health.

### Themes of content analysis

#### Health-disease processes

Our results address the health-disease process based on the dialogue of the interviewed women agriculturalists at RDS Rio Negro, exploring elements that constitute this complex dynamic. Participants shared experiences related to seeking medical care, emphasizing the importance of physical and mental health in their daily activities. Agriculture emerged as a key factor for food security and health promotion by ensuring access to nutritious food, physical activity, and cultural continuity. Crucially, it provides autonomy and financial independence, which directly support mental health and social dignity. Several women emphasized that without their work, they would be unable to afford basic items such as food and medicine, underscoring how agricultural labor is essential to their physical survival and emotional well-being.

Participants’ narratives emphasized diet, home remedies, and resilience, reflecting a holistic view of health. This study highlights the need to consider multiple factors in understanding health in context. RDS Rio Negro leaders noted increased mental health issues, domestic and sexual violence, and malnutrition in nearby communities during the COVID-19 pandemic. The interviews also showed health risks tied to agricultural labor, including snake bites, wild animal attacks, boat accidents, and injuries. Women reported arm pain from repetitive tasks, back pain from posture or lifting, and general physical exhaustion.

#### Treatment: traditional treatment and basic healthcare

Women agriculturalists prioritize home remedies - mainly plants, animal parts, and honey - as their first response to illness. Although they use conventional medicine, limited access to physicians and drugs makes traditional practices a culturally rooted and low-cost option. Medical care is sought only when conditions worsen due to the high cost and effort of traveling to health centers or hospital. Remedies mostly consist of teas and ointments made from plants, although some may include harmful substances such as kerosene, gasoline, or diesel, as noted by interviewees.

In the RDS Rio Negro, the only basic health unit (BHU) is in the Nossa Senhora do Perpétuo Socorro community, serving 14 communities. Its team includes a physician, a nurse, dental staff, and community health agents. A Telehealth point by the Amazon Sustainable Foundation supports remote care, reducing the need for travel. The unit operates from Tuesday to Thursday and is run by the Iranduba Municipal Health Department (SEMSA, acronym in Portuguese). However, many families require home visits due to financial constraints and distance, since some live over an hour away.

Trust-based relationships - fostered by listening, support, and open dialogue - are key to encouraging treatment adherence and care-seeking. These “light care technologies” help professionals understand local realities. Notably, the only physician in the area was a foreign participant in the federal program called “More Doctors”.

Beyond the health unit, missionary boats offer medical, dental, and psychological care. Other services include those from riverine BHU, boats accommodating river family health teams to serve the riverine population of the Legal Amazon. Nevertheless, service gaps remain. In 2021, the SEMSA team reported canceling Wednesday home visits due to fuel shortages. Due to these limitations, residents often seek care outside the RDS Rio Negro.

#### Sustainable interconnections: cross-cutting dialogues on work, agriculture, gender, and environment

According to women agriculturalists in the RDS Rio Negro, work promotes health, individual and collective autonomy, and identity. Some feel recognized by their community or outsiders, whereas others say their work lacks proper acknowledgment, especially from the government. They stress the need for effective funding policies. These women value agricultural work for personal and communal subsistence and find creative solutions to daily challenges. In the absence of inputs, they may hire help for land clearing, improvise greenhouses with wood and thatch, or buy polished *açaí* seeds when equipment is unavailable. Interviewees also noted gender-based labor divisions: men often avoid domestic tasks, which are seen as women’s responsibility unless the women are absent. The labeling of strength-based tasks as masculine reinforces gender norms.

Findings show that work and environment are key to health, yet both are under threat. Participants reported invasions, land grabbing, and corruption among local leaders. Residents say entrepreneurs from Manaus (capital of the state) are illegally acquiring land for development. Iranduba, covering 80% of the RDS Rio Negro and near Manaus, is central to such conflicts, especially since the 2011 Rio Negro Bridge spurred real estate speculation. Community leaders have reportedly offered land to outsiders and facilitated access.

One farmer reported her leader’s illegal land sales and refusal to provide documents to residents who refuse to pay association fees, even selling their documents. Such insecurity affects land-based work due to the risk of expulsion, deforestation, and environmental harm. Despite established rules, illegal activities persist, threatening local ways of life. While some exploit the reserve, women agriculturalists see nature as life and sustenance, deeply tied to their identity and well-being.

Women agriculturalists also show a significant reluctance toward city life, which can be attributed to a range of stressful factors in urban environments, such as robberies, crime, pollution, noise, and inadequate sanitation. These factors are also linked to the invasion and land grabbing process in the RDS Rio Negro following the construction of the bridge on Rio Negro River (Figure 1). These findings underscore the importance of preserving their environment to maintain their way of life and overall well-being.

## Discussion

Our results showed that the health-disease process among women agriculturalists in the central Amazon goes beyond the traditional view that focuses solely on the biological aspects of illness. It considers factors such as lifestyle, socioeconomic conditions, access to healthcare, physical and social environment, genetics conditions of the populations, health behaviors, mode of production, and other factors. An example refers to the pressure from large projects geared toward the capitalist model of society, which has harm traditional communities in the Amazon, affecting their sustainability and way of life ^47^ and increasing public health disparities ^48^. Therefore, our results show the health-disease process is regarded as a multifactorial and complex phenomenon. In this scenario, the relentless pursuit of profit often results in forced displacement ^26^, the loss of natural resources ^7^, cultural disintegration ^6^ ^,^ ^11^ ^,^ ^19^ ^,^ ^26^, and negative impacts on the health of these communities, such as the increase in endemic diseases ^48^. In the context of the central Amazon, this comprehensive perspective underscores the challenges these traditional communities face ^4^.

Resoluteness, a principle of Brazilian Unified National Health System (SUS, acronym in Portuguese), states that healthcare services must be able to address users’ needs and community health issues ^49^. Our findings show the need to strengthen local primary care. Shortages of inputs and personnel reduce team performance and burden neighboring municipalities ^50^ ^,^ ^51^. Resolving these issues requires open communication and active listening by healthcare providers since treatment adherence depends on user engagement ^52^. Interdisciplinary teams are key to transforming care, benefiting users and providers, and restructuring healthcare delivery ^53^. Teamwork encourages users’ participation in decisions, acknowledging them as social subjects shaped by history, culture, and environment ^54^ ^,^ ^55^.

Ongoing training is vital for rural professionals, especially in the Amazon, to develop intercultural sensitivity and align care with local ways of life ^21^ ^,^ ^25^. In addition to strengthening teams, expanding primary care in the RDS Rio Negro is urgent. The area has only one BHU. This expansion can include formal basic health units and informal points (churches, community centers) ^55^, ensuring universal access to the SUS ^49^. Health integration with environmental and sanitation actions is also critical as many communities lack treated water, leading to preventable diseases ^56^. *Law n. 8,080/1990* highlights the role of epidemiology in setting priorities and guiding health programs ^49^.

Given the biodiversity and rural labor in the Amazon, another concern refers to the rise in accidents involving venomous animals, now common in farmers’ daily lives ^57^. Personal protective equipment use and preventive guidance must be promoted ^58^ as accidents may lead to health issues and loss of income and autonomy for women agriculturalists, whose work is tightly linked to their well-being. Our data also show that work, agriculture, environment, and gender intersect in ways that are threatened by land invasions in the Amazon ^8^ ^,^ ^9^. These disrupt not just space but community bonds. Environmental degradation worsens health inequalities ^4^ ^,^ ^45^, as per this study. Large infrastructure projects such as bridges and highways often worsen these conditions, degrading ecosystems and traditional livelihoods ^6^ ^,^ ^7^ ^,^ ^8^ ^,^ ^9^ ^,^ ^35^ ^,^ ^59^. We observed growing mental health demands during the COVID-19 pandemic and increased zoonotic spillover risks due to large projects ^20^ ^,^ ^48^ ^,^ ^60^ ^,^ ^61^ ^,^ ^62^ ^,^ ^63^. These impacts extend beyond the pandemic, intensifying violence against women and children and raising malnutrition cases. Women agriculturalists are especially vulnerable, as in Altamira after the Belo Monte Hydroelectric Plant impacts ^64^ ^,^ ^65^.

In the central Amazon, the paving of BR-319 highway and oil and gas projects in the Amazon Basin and Solimões Sedimentary Basin may worsen health inequalities in traditional areas ^60^ ^,^ ^61^. These initiatives must be reconsidered due to their effects on the environment, regional sustainability, epidemic risk, and global climate change ^60^ ^,^ ^61^. The climate crisis, as experienced in the Amazon in 2023 by the impacts of El Niño, climate change ^36^, and the forest tipping point ^66^, will exacerbate these hardships. This is evident in the increased isolation of riverside communities due to drought, reducing access to clean water and basic healthcare ^36^. In the Brazilian Amazon, severe droughts have interrupted river navigation for weeks or even months, isolating more than 6,000 Indigenous and non-Indigenous localities and limiting access to food, medical supplies, education, and essential healthcare services and potable water ^36^. As highlighted in this study, the isolation of communities during drought further compounds the challenges associated with accessing fundamental healthcare services.

The climatic anomalies contributing to the crisis in the region are likely to extend to central Amazonia if the BR-319 highway is paved ^67^. This extension could amplify health-disease processes due to zoonoses and epidemiological outbreaks ^48^ ^,^ ^68^ and isolation due to an increased frequency of drought events leading to shortages in food, medical supplies, healthcare providers, and potable water ^36^. The migration of actors linked to land invasions facilitated by the BR-319 highway, such as land grabbers, gold miners, and timber companies, would also escalate deforestation in the region, intensifying the pressure on areas traditionally used for social well-being, work, and safety ^7^ ^,^ ^8^ ^,^ ^9^. In studies conducted in a hotspot of deforestation in this area, land prices varied from BRL 20 (USD 4.86) to BRL 3,000 (USD 729.92) per hectare, depending on the accessibility of these areas ^9^. The region has also showed a complete lack of governance and collusion among regulatory authorities regarding issues such as illegal deforestation and land encroachment ^7^. With the potential paving of a highway through the central Amazon, this trend is expected to reach the northern region via BR-174 and the western Amazon through the planned AM-366, crossing the Purus River ^7^ ^,^ ^8^ ^,^ ^9^ ^,^ ^16^ ^,^ ^17^ ^,^ ^61^.

According to the interviewed women, land speculation threatens their workspace and harms their health as their well-being depends on their ability to work. In the Ramal do Uga-Uga region of the RDS Rio Negro, interviews showed a growing real estate network that expanded after the Rio Negro Bridge linked the RDS Rio Negro to Manaus ^35^. Although the RDS Rio Negro is a Conservation Unit managed by Amazonas State Environmental Agency, travel along highways AM-070 and AM-352 shows illegal roads and a lack of effective oversight ^35^. In this manner, contrary to what politicians argue in the Amazonian region ^48^ ^,^ ^61^, highways and bridges fail to minimize health disparities, substantially increasing health-related challenges across the Amazon, surpassing the capacity of states and federal government to meet local healthcare demands ^61^. The exacerbation of the climate crisis due to deforestation caused by these projects would disproportionately worsen drought events ^16^ ^,^ ^68^, restricting access to healthcare and supplies to interior traditional communities ^36^. This, coupled with pressure on territories from invaders ^7^ ^,^ ^8^ ^,^ ^9^, would increase climate refugees and land conflicts, potentially endangering traditional communities in the central Amazonia and their way of life.

## Conclusion

Our study highlights the complex and multifactorial nature of the health-disease process among women agriculturalists in traditional communities of the central Amazon. It underscores the importance of adopting a holistic perspective that integrates biological, socioeconomic, lifestyle, environmental, genetic, and behavioral dimensions. Within this framework, large infrastructure projects - such as roads and bridges - emerge as aggravating factors as they disrupt local ecosystems, stimulate land grabbing, and heighten insecurity, all of which directly impact these women’s health and well-being. Neglecting these findings exacerbates existing health vulnerabilities and fuels a chain of associated problems, including rising rates of domestic violence, sexual violence against children, and malnutrition. Therefore, protecting these communities requires more than preserving their territory; it demands the defense of their health, dignity, and cultural heritage.

## Data Availability

The research data are available upon request to the corresponding author.
